# Parameters of glucose metabolism and the aging brain: a magnetization transfer imaging study of brain macro- and micro-structure in older adults without diabetes

**DOI:** 10.1007/s11357-015-9802-0

**Published:** 2015-07-17

**Authors:** Abimbola A. Akintola, Annette van den Berg, Irmhild Altmann-Schneider, Steffy W. Jansen, Mark A. van Buchem, P. Eline Slagboom, Rudi G. Westendorp, Diana van Heemst, Jeroen van der Grond

**Affiliations:** Department of Gerontology and Geriatrics, Leiden University Medical Centre, Leiden, the Netherlands; Department of Radiology, Leiden University Medical Centre, Leiden, the Netherlands; Department of Molecular Epidemiology, Leiden University Medical Centre, Leiden, the Netherlands; Department of Public Health, University of Copenhagen, Copenhagen, Denmark; Netherlands Consortium for Healthy Ageing, Leiden, the Netherlands

**Keywords:** Insulin, Glucose, MRI, Magnetization transfer imaging, Brain, Aging

## Abstract

Given the concurrent, escalating epidemic of diabetes mellitus and neurodegenerative diseases, two age-related disorders, we aimed to understand the relation between parameters of glucose metabolism and indices of pathology in the aging brain. From the Leiden Longevity Study, 132 participants (mean age 66 years) underwent a 2-h oral glucose tolerance test to assess glucose tolerance (fasted and area under the curve (AUC) glucose), insulin sensitivity (fasted and AUC insulin and homeostatic model assessment of insulin sensitivity (HOMA-IS)) and insulin secretion (insulinogenic index). 3-T brain MRI was used to detect macro-structural damage (atrophy, white matter hyper-intensities, infarcts and/or micro-bleeds) and magnetization transfer imaging (MTI) to detect loss of micro-structural homogeneity that remains otherwise invisible on conventional MRI. Macro-structurally, higher fasted glucose was significantly associated with white matter atrophy (*P* = 0.028). Micro-structurally, decreased magnetization transfer ratio (MTR) peak height in gray matter was associated with higher fasted insulin (*P* = 0.010), AUC_insulin_ (*P* = 0.001), insulinogenic index (*P* = 0.008) and lower HOMA-IS index (*P* < 0.001). Similar significant associations were found for white matter. Thus, while higher glucose was associated with macro-structural damage, impaired insulin action was associated more strongly with reduced micro-structural brain parenchymal homogeneity. These findings offer some insight into the association between different parameters of glucose metabolism (impairment of which is characteristic of diabetes mellitus) and brain aging.

## Introduction

The rising prevalence of type 2 diabetes and neurodegenerative disease over the past several decades has made it of critical importance to understand the relation of glucose and insulin with the aging brain. The prevalence of type 2 diabetes steadily increases with age, with estimates suggesting that more than half of individuals older than 65 years have either diabetes or pre-diabetes (Cowie et al. 2006). Diabetes and pre-diabetic states, characterized by impairments in glucose, insulin and insulin sensitivity, are known to be risk factors for cognitive decline, mild cognitive impairment and dementia (Biessels et al. 2006; Cowie et al. 2006). It is also known that higher glucose levels, in the absence of (pre-) diabetes, are associated with increased risk of accelerated cognitive decline (Vagelatos and Eslick 2013) or dementia (Crane et al. 2013) in older persons. Furthermore, there is a surge of new information pointing towards high circulating insulin and insulin resistance as mediators of neurodegenerative brain diseases (de la Monte and Wands 2008; Steen et al. 2005). It however remains unclear what the association is of ‘normal’ glucose and insulin (glucose and insulin levels within the population reference range), with macro- and micro-structural brain changes in older persons without diabetes.

**Fig. 1:**
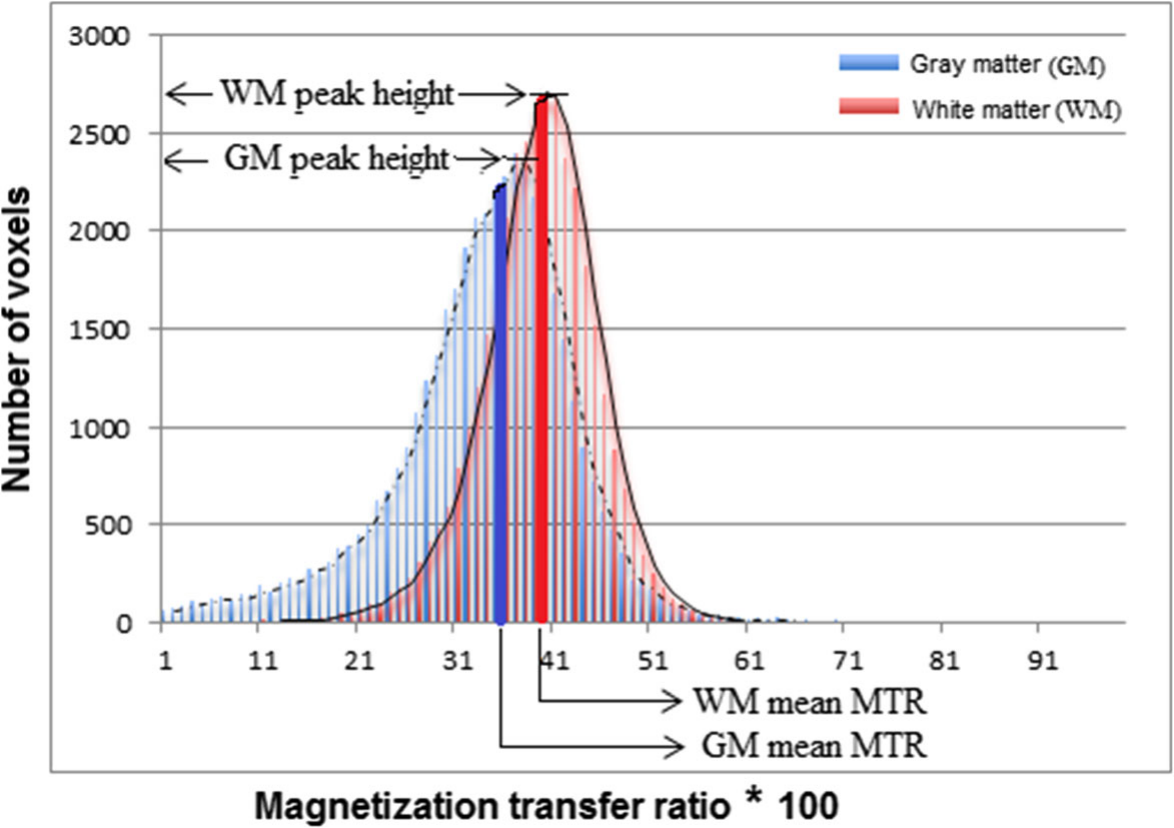
MTR histogram of gray and white matter showing mean MTR and MTR peak height. MTR histograms of both gray matter (in *blue*) and white matter (in *red*) with *trend lines* (moving averages). The MTR peak height, the highest peak of each histogram, is defined as the number of voxels with the most frequent MTR value. The mean MTR, shown as the *thick vertical blue line* (for gray matter) and *red line* (white matter) is defined as the average of the MTR value of all voxels in the region(s) of interest

Existing literature has demonstrated a decrease in total brain volume in relation to diabetic and pre-diabetic states in late middle age (Tan et al. 2011). Besides these macro-structural changes, however, micro-structural changes may possibly occur in normal appearing brain tissue in relation to glucose and insulin, serving as indices of brain pathology. These micro-structural brain tissue changes, which are beyond the spatial resolution of the conventional magnetic resonance imaging (MRI), can be detected with magnetization transfer (MT) imaging (MTI) (Inglese and Ge 2004). MTI can also detect differences in the degree of tissue destruction in macro-structural lesions (cerebral atrophy, white matter hyper-intensities, lacunar infarcts or cerebral micro-bleeds in the white matter). MTI is based on the exchange of magnetization between protons bound to macro-molecules and protons of free water molecules inside tissue. The MT ratio (MTR), which reflects the scale of this exchange, has been shown to decrease in the presence of brain tissue damage due to pathology or aging (Benedetti et al. 2006; Sala et al. 2014). MTR is calculated per voxel with subsequent generation of a histogram per region of interest, from which mean MTR and MTR peak height can be determined. The highest peak of each histogram is the MTR peak height and is defined as the number of voxels with the most frequent MTR value. The mean MTR is defined as the average of the MTR value of all voxels in the region(s) of interest, as depicted in Fig. 1. The peak height of a MTR histogram represents the uniformity within the region of interest. Both mean MTR and peak height reflect different aspects of MTR, and they may show different sensitivity in detecting structural changes in the brain. Specifically, MTR peak height has been suggested to be a relatively specific quantitative measure of micro-structural brain parenchymal abnormalities, including myelin content and axonal numbers (Rademacher et al. 1999). A lower brain tissue MTR peak height indicates loss of homogeneity of brain tissue (van Buchem and Tofts 2000) and is observed in brain parenchymal abnormalities that develop with aging or disease.

The aim of the present study was to investigate the association between parameters of glucose metabolism and three indices of brain pathology, namely gray and white matter parenchymal volumes and atrophy, macroscopic brain damage and micro-structural integrity. Parameters of glucose metabolism were derived from oral glucose tolerance test (OGTT) (Takeuchi et al. 2000), and included measures of glucose tolerance, insulin sensitivity and pancreatic β-cell secretory capacity. Macro-structural brain parameters were measured using MRI and included the presence and number of micro-bleeds, lacunar infarcts and/or volume of white matter hyper-intensities. Micro-structural parameters included quantification of homogeneity of brain parenchyma, including axonal and myelin integrity, as measured using MTI-derived mean MTR and peak height.

## Materials and methods

### Study population

Subjects were included from the Leiden Longevity Study (LLS), which was set up to investigate determinants and pathways associated with healthy aging and longevity, as previously described (Schoenmaker et al. 2006). A total of 421 Caucasian families were included and regarded as enriched for familial longevity if at least two long-lived siblings were alive and fulfilled the age criteria of ≥89 years for males or ≥91 years for females. Sex-specific age criteria were used due to the higher life expectancy of females compared to males. No selection criteria on health or demographic characteristics were applied. Offspring of these long-lived nonagenarians were also included, having inherited on average 50 % of the genetic propensity of their long-lived parent. Partners of these offspring, with whom they have shared the same socio-economic and geographical environment for decades and who are of a similar age, were also enrolled. In total, 2415 offspring and partners were included in LLS.

Of the 2415 subjects included in LLS, a random subset of 370 underwent a MRI scan of the brain, as had been previously described (Altmann-Schneider et al. 2012). Of the 370 subjects, 132 non-diabetic subjects (75 offspring and 57 partners, comprising 49 couples) also underwent OGTT. Therefore, a total of 132 non-diabetic subjects with reliable, complete OGTT, brain MRI and MTI data were included in this study. These participants had no known history of dementia, were free of memory complaints, and had never visited a memory clinic. Furthermore, the participants underwent three cognitive tests—Stroop Color Word test (Stroop test), to test for cognitive flexibility and executive function; Digit Symbol Substitution Test (DSST), to evaluate attention and processing speed; and 15-Picture Word Learning Test (15-PLT), to test for immediate and delayed memory, as had been earlier described (Stijntjes et al. 2013). For Stroop test, subjects were asked to read a color name, which was displayed in a color different from the color that it actually names. The outcome parameter was the time (seconds) needed to complete the test; a higher score indicates a worse performance. For the DSST, the participants had to match certain digits with letters according to a provided key. Outcome parameter was the number of correct digit-symbol combinations within 90 s. For 15-PLT, 15 pictures were successively presented at a rate of 1 per 2 s after which the subject was asked to recall as many pictures as possible. This procedure was carried out three times (PLT-1, PLT-2 and PLT-3). After 20 min, delayed recall was tested. Outcome parameters were the number of correct pictures after each trial for PLT-immediate (immediate recall) and after 20 min for PLT-delayed (delayed recall).

The Medical Ethical Committee of the Leiden University Medical Centre approved the study and written informed consent was obtained from all participants.

### OGTT-derived parameters of glucose metabolism

In the morning after an overnight fast of at least 10 h, an OGTT was performed with a 75-g glucose load per 300 mL of water. Venous blood samples were withdrawn at 0, 30, 60 and 120 min after oral ingestion of the glucose load. Parameters derived from the OGTT included fasted glucose and area under the curve (AUC) for glucose (AUC_glucose_), which are measures of glucose tolerance; fasted insulin, AUC_insulin_ and HOMA-IS, which are measures of insulin action and sensitivity (Matthews et al. 1985); and insulinogenic index (Hanson et al. 2000) which is a measure of pancreatic beta cell secretory capacity. AUC for glucose (AUC_glucose_) and insulin (AUC_insulin_) were calculated using the trapezoid formula. The glucose and insulin curve was first divided into a number of strips of equal width. Then, the area of the trapezium formed approximated the area of each strip. The sum of these approximations gave the final numerical result of the area under the glucose (AUC_glucose_) and insulin (AUC_insulin_) curves, taking into account the measurements themselves and the time distance between the measurements (Pruessner et al. 2003). HOMA-IS was calculated by dividing 22.5 by the product of the fasting levels of serum insulin (in mU/L) and glucose (in mmol/L) (Matthews et al. 1985). Insulogenic index was calculated by dividing increments of insulin at 30 min compared to fasting values by the corresponding increment at 30 min of glucose levels compared to fasted glucose values (Hanson et al. 2000).

#### Biochemical analysis

All serum measurements were performed using fully automated equipment. For glucose, the Hitachi Modular P 800 from Roche (Almere, the Netherlands) was used, with coefficient of variation (CV) less than 5 %. For insulin levels, the Immulite 2500 from DPC (Los Angeles, CA) was used, with CV of less than 8 %.

### Brain MRI study

#### MRI acquisition

All imaging were performed on a whole body MR system operating at 3-T field strength (Philips Medical Systems, Best, The Netherlands). Three-dimensional (3D) T1-weighted (repetition time 9.7 ms, voxel size 1.17 × 1.17 × 1.4 mm, covering the entire brain, acquisition time ≈5 min), T2-weighted (repetition time 4200 ms, matrix size 448 × 320, 40 transverse slices with slice thickness of 3.6 mm, covering the entire brain) were acquired. Furthermore, fluid-attenuated inversion recovery (FLAIR, repetition time 11 000 ms, matrix size 320 × 240, 25 transverse slices with slice thickness of 5 mm covering the entire brain), T2*-weighted images (repetition time 45 ms, field of view 250 × 175 × 112 mm) and MTI images were acquired. MTI was performed with the following parameters: TR = 100 ms, TE = 11 ms, FA = 9°, FOV = 224 × 180 × 144 mm, matrix size 224 × 169, and 20 slices with a 7-mm thickness.

#### Image processing and analysis

Using the Functional MRI of the Brain (FMIRB) Software Library (FSL) tools, the various analytical techniques and tools that were used for processing and analysis of the MRI scans are described below.

#### Brain volumes

Whole brain, gray matter and white matter volumes were calculated using the FSL-tool Structural Image Evaluation, using Normalization, of Atrophy (SIENAX) (Smith 2002). SIENAX extracted brain and skull images from the single whole-head input data (Jenkinson et al. 2002). Thereafter, tissue-type segmentation with partial volume estimation was performed using FMRIB’s automated segmentation tool (FAST), and total volume of brain tissue, including separate estimates of volumes of gray matter and white matter, was obtained. (Zhang et al. 2001). Additionally, hippocampal volume was calculated using the FMRIB’s Integration Registration and Segmentation Tool (FIRST), as has been previously described (Altmann-Schneider et al. 2012).

#### Brain atrophy

Atrophy was defined as the difference between intracranial and brain volume divided by intracranial volume multiplied by hundred percent. Using FSL, an estimate for the total intracranial volume was obtained by linearly aligning each subject’s brain to the MNI152 space and computing the inverse of the determinant of the affine matrix.

#### White matter hyper-intensities, lacunar infarcts and cerebral micro-bleeds

Medical Image Processing, Analysis, and Visualization (MIPAV) software was used to visualize the MRI scans. WMHs and lacunar infarcts were evaluated using FLAIR, T2-weighted and 3-D T1-weighted images. Analysis was done blinded to age, sex and subject identity.

White matter hyper-intensities (WMHs) were defined as areas within the cerebral white matter with increased signal intensity on both FLAIR and T2-weighted images, without mass effect (i.e. the increased signal intensities were not secondary to pushing or displacing by surrounding tissue). Measurement of the WMH volume was carried out using the automated method, whereby 3DT1-weighted images were skull stripped (Smith et al. 2002) and the FLAIR and 3DT1 image were co-registered in order to create a brain-extracted FLAIR image (Jenkinson et al. 2002). This brain-extracted FLAIR image was subsequently affine registered to MNI152 standard space using the FMRIB’s Linear Image Registration Tool. A conservative MNI152 standard space white matter mask was used to extract the white matter from the FLAIR image. Finally, after excluding the cerebellum and brainstem, a threshold was set to identify which white matter voxels were hyper-intense, followed by manually checking and editing for quality control.

Lacunar infarcts were defined as parenchymal defects within the cerebral white matter not extending into the cortical gray matter, with signal intensity centrally corresponding to that of cerebral spinal fluid on all three imaging sequences, surrounded by a rim of increased signal intensity on FLAIR (Longstreth et al. 1998). Lacunar infarct diameter was defined to be >2 mm. To distinguish lacunar infarcts from normal dilated perivascular spaces (Virchow-Robin-Spaces), hyper-intensities within the lower one third of the corpus striatum of the basal ganglia and a diameter of <2 mm were excluded (Bokura et al. 1998).

Cerebral micro-bleeds (CMBs) were defined as round focal areas of signal void on T2-weighted images, which increased in size on T2*-weighted images (blooming effect) (Greenberg et al. 2009). Thus, CMBs can be distinguished from look-alikes such as vascular flow voids. Symmetric hypo-intensities in the basal ganglia were disregarded, as they are likely to represent calcifications or non-hemorrhagic iron deposition (Greenberg et al. 2009).

#### MTI data processing

The individual 3DT1 images were skull stripped using BET (brain extracting tool) and segmented using FAST (FMRIB’s automated segmentation tool), resulting in individual brain masks for white matter and cortical gray matter. Subsequently, non-saturated (M0) and saturated images were registered to the T1 image, using FMRIB’s linear image registration tool (FLIRT). Registration matrices from the previous step were used to co-register the non-saturated M0 images and the individual brain masks for gray and white matter to create separate gray and white matter MTR maps. To correct for possible partial volume effects, an eroded mask of these segmentations was created by removing one voxel in plane for both volumes of interest (VOIs). Individual MTR maps were calculated voxel by voxel following the equation MTR = (M0 − M1) / M0 and MTR histograms were generated for both VOIs. Mean MTR, MTR peak height, corrected for the size of the VOI, and MTR peak location were calculated from each MTR histogram. The mean of the MTR value of all voxels in the histogram is the mean MTR, and the highest peak of each histogram is the MTR peak height, as depicted in Fig. 1.

For the voxel-based analysis of gray matter, the MTR GM maps were aligned to MNI standard space using non-linear transformation (Andersson et al. 2007) and averaged to create a reference template for MTR images. Then, all individual gray matter MTR maps were non-linearly registered to this template, divided by the Jacobian of the warp field and smoothed with an isotropic Gaussian kernel with a sigma of 3 mm (Cosottini et al. 2011).

### Statistical analyses

Analyses were conducted in a three-step approach. First, we assessed the association between OGTT-derived parameters of glucose metabolism and brain atrophy. Secondly, we investigated the association between OGTT-derived parameters and macroscopic brain damage (cerebral micro-bleeds, lacunar infarcts and volume of white matter hyper-intensities). Thirdly, we assessed the relation between OGTT-derived parameters and brain micro-structural integrity.

Data analysis was done using Statistical Package for Social Sciences (SPSS) software for windows (version 20.0). Unless otherwise stated, data are presented as mean with standard deviation (SD). Distributions of continuous variables were examined for normality, logarithmically transformed when appropriate and used in calculations. Serum insulin levels (fasted and AUC_insulin_) and HOMA-IS were logarithmically transformed with resultant normalization of their skewed distribution. Geometric means are reported for transformed variables. Linear regression model was used to investigate the associations between OGTT-derived parameters, gray and white matter atrophy and MR brain tissue markers for micro-structural integrity. The initial analyses were adjusted for age, sex and descent (Leiden longevity offspring/partner status). Extended models further included smoking status, BMI, use of anti-hypertensive and use of lipid-lowering agents. Statistical significance was defined as *P* < 0.05.

For MRI data, voxel-wise analysis statistics was carried out with FSL randomize using permutation-based non-parametric testing (5000 permutations). Threshold-free cluster enhancement was used to optimize sensitivity to different shapes and sizes of MRI signals, to separate true signals from noise (Smith and Nichols 2009), with a significance level set at *P* < 0.05, controlled for family wise error rate. Age and gender of participants were inserted as covariates in the model.

## Results

Characteristics of the study subjects are summarized in Table 1. The mean age of the subjects was 66 years (SD 6.6); 62 (47 %) were males and 70 (53 %) were females. Medical history showed that 29 % had hypertension, 22 % used anti-hypertensive medication(s), 14 % used lipid-lowering drugs, 1 % had had a previous CVA and 1 % had had a previous myocardial infarction. All OGTT-derived parameters were within normal reference range. Table 1 also shows the mean gray and white matter volumes and atrophy, and the mean MTI parameters of the subjects. The mean time needed to complete the Stroop test was 48 s, while there was an average of 47 correct answers for the DSST. Mean number of correct pictures for 15-PLT immediate and delayed recall was 10 and 11, respectively (Table 1). From the cross-sectional data, no significant correlation was found between the cognitive tests, which are a measure of functional brain integrity, and MTR peak height, which is a measure of micro-structural brain parenchymal tissue homogeneity, nor with white matter hyper-intensities, lacunar infarcts or cerebral micro-bleeds (data not shown).

**Table 1:** Description of study subjects

Characteristics	*N* = 132
Demographics	
Men, *n* (%)	62 (47)
Age in years	66 (6.6)
BMI in kg/m^2^	26 (4)
Current smoking, *n* (%)	11 (8)
Medical history	
Myocardial infarct, *n* (%)	1 (0.8)
Hypertension, *n* (%)	29 (22)
Use of anti-hypertensive medications, *n* (%)	37 (28)
CVA, *n* (%)	1 (0.8)
Use of lipid-lowering medications, *n* (%)	14 (11)
OGTT-derived characteristics	
Fasted glucose in mmol/L	5.1 (0.6)
AUC glucose in mmol/L	14 (4)
Fasted insulin in pmol/L, median (25th, 75th percentile)	42 (28, 73)
AUC insulin, median (25th, 75th percentile)	94 (64, 139)
HOMA-IS index, median (25th, 75th percentile)	−1.5 (−0.7, −2.4)
Insulinogenic index, median (25th, 75th percentile)	13 (7, 20)
HbA1c in % (mmol/mol)	5.2 (33)
Cognitive tests results	
Digit Symbol Substitution Test, correct answers	46.5 (10)
Stroop test, seconds	48 (13)
15-PLTi, correct pictures	10 (2)
15-PLTd, correct pictures	11 (2)
Brain volumes in cm^3^	
White matter	541 (57)
Gray matter	542 (36)
Brain atrophy in %	
Whole brain	24.1 (3.4)
White matter	2.8 (5.4)
Gray matter	20.9 (4.5)
Mean magnetization transfer ratio	
White matter	0.385 (0.010)
Gray matter	0.333 (0.097)
Peak height, pixel count × 10^3^	
White matter	117 (24)
Gray matter	74 (12)
Macro-structural characteristics	
White matter hyper-intensities, *n* (%)	119 (90)
Lacunar infarcts, *n* (%)	5 (3.8)
Cerebral micro-bleeds, *n* (%)	14 (11)

Values are means (*SD*, standard deviation), unless otherwise stated. Age refers to age at MRI examination. *BMI* body mass index, *CVA* cerebrovascular accident, *OGTT* oral glucose tolerance test, *AUC* area under the curve, *HOMA-IS* homeostatic model assessment of insulin sensitivity, *15-PLTi* 15-Picture Learning Test-immediate recall, *15-PLTd* 15-Picture Learning Test-delayed recall

### Parameters of glucose metabolism and atrophy

We assessed the association of OGTT-derived parameters of glucose metabolism with gray matter, white matter and hippocampal atrophy. Parameters of glucose metabolism included measures of glucose tolerance (fasted glucose and area under the glucose curve (AUC_glucose_)), measures of insulin sensitivity (fasted insulin, area under the insulin curve (AUC_insulin_) and homeostatic model assessment of insulin sensitivity (HOMA-IS) index) and a measure of pancreatic β-cells secretory capacity (insulinogenic index). As shown in Table 2, higher fasted glucose was associated with white matter atrophy (*β* = −0.189, *P* = 0.028). No association was found between any of the OGTT-derived parameters and hippocampal atrophy (data not shown) or gray matter atrophy (Table 2).

**Table 2:** Association of gray and white matter atrophy with parameters of glucose metabolism

	Atrophy					
	Gray matter			White matter		
	Beta	*P* value	*R* ^2^	Beta	*P* value	*R* ^2^
Fasted glucose	0.007	0.924	0.336	−0.189	0.028*	0.191
AUC glucose	−0.042	0.586	0.337	−0.148	0.084	0.179
Fasted insulin	0.002	0.983	0.336	−0.082	0.325	0.166
AUC insulin	−0.059	0.420	0.339	−0.076	0.361	0.165
HOMA-IS index	−0.003	0.968	0.336	0.105	0.213	0.170
Insulinogenic Index	−0.076	0.302	0.342	0.087	0.292	0.167

Atrophy is defined as the difference between intracranial and brain volumes divided by intracranial volume multiplied by hundred percent. All insulin values were log-transformed. Associations are expressed as standardized Beta with corresponding *P*-values. Results are from linear regression analysis corrected for age, gender and offspring- partner status

*AUC* Area under the curve, *HOMA-IS* Homeostatic model assessment of insulin sensitivity

*p < 0.05

### Parameters of glucose metabolism and macroscopic brain damage

The associations of measures of glucose tolerance, measures of insulin action and insulinogenic index with macroscopic brain damage were investigated (data not shown). Indices of macroscopic brain damage included the presence and number of micro-bleeds, lacunar infarcts and/or volume of the white matter hyper-intensities, as measured using MRI. Fasted glucose was inversely associated with number of cerebral micro-bleeds (*β* = −0.214, *R*^*2*^ = 0.053, *P* = 0.045). None of the other OGTT-derived parameters (AUC_glucose_, fasted insulin AUC_insulin_, HOMA-IS or insulinogenic index) were significantly associated with indices of macroscopic brain damage. Repetition of the analyses while adjusting for age, gender, descent, smoking status, use of anti-hypertensive and use of lipid-lowering agents did not materially change the results.

### Parameters of glucose metabolism and brain micro-structure

Table 3 shows the association of OGTT-derived glucose and insulin parameters with micro-structural gray and white matter parenchymal integrity, measured via magnetization transfer imaging, and expressed in mean MTR and MTR peak height. A lower brain tissue mean MTR or peak height indicates loss of homogeneity of brain tissue or tissue damage. In the gray matter, parameters of reduced insulin action, namely, higher fasted insulin (*β* = −0.213, *P* = 0.010), AUC_insulin_ (*β* = −0.276, *P* = 0.001), insulinogenic index (*β* = −0.289, *P* < 0.001) and decreased HOMA-IS (*β* = 0.220, *P* = 0.008), were significantly associated with lower gray matter MTR peak height. Similarly, higher AUC_insulin_ was associated with reduced mean gray matter MTR. Similar trends were seen between other OGTT-derived parameters and mean MTR, but these did not reach statistical significance.

**Table 3:** Association of magnetization transfer imaging (MTI)-derived integrity of gray and white matter micro-structure with parameters of glucose metabolism

	Gray matter						White matter					
	Mean MTR			Peak height			Mean MTR			Peak height		
	Beta	*P* value	*R* ^2^	Beta	*P* value	*R* ^2^	Beta	*P* value	*R* ^2^	Beta	*P* value	*R* ^2^
Fasted glucose	−0.124	0.166	0.159	−0.150	0.084	0.216	0.058	0.528	0.106	−0.050	0.572	0.181
AUC glucose	−0.165	0.063	0.170	−0.113	0.191	0.207	−0.031	0.737	0.103	−0.133	0.127	0.194
Fasted insulin	−0.068	0.432	0.150	−0.213	0.010*	0.239	0.110	0.213	0.114	−0.189	0.024*	0.213
AUC insulin	−0.181	0.033*	0.177	−0.276	0.001*	0.269	−0.033	0.709	0.104	−0.264	0.001*	0.246
HOMA-IS index	0.081	0.351	0.152	0.220	0.008*	0.241	−0.110	0.217	0.114	0.184	0.030*	0.210
Insulinogenic index	−0.151	0.075	0.168	−0.289	<0.001*	0.277	0.018	0.833	0.103	−0.210	0.011*	0.221

All insulin values were log transformed. The standardized beta and corresponding *P* values are shown for analysis using individual brain volume corrected for head size. Results are from linear regression analysis corrected for age, gender and offspring partner status

*MTR* magnetization transfer ratio, *AUC* area under the curve, *HOMA-IS* homeostatic model assessment of insulin sensitivity

*p < 0.05

In the white matter, increased fasted insulin (*β* = −0.189, *P* = 0.024), AUC_insulin_ (*β* = −0.264, *P* = 0.001), insulinogenic index (*β* = −0.210, *P* = 0.011) and decreased HOMA-IS (*β* = 0.184, *P* = 0.030) were significantly associated with decreased white matter MTR peak height. These associations did not materially change after adjustment for age, gender, descent, smoking status, BMI, use of anti-hypertensive and use of lipid-lowering agents.

**Fig. 2:**
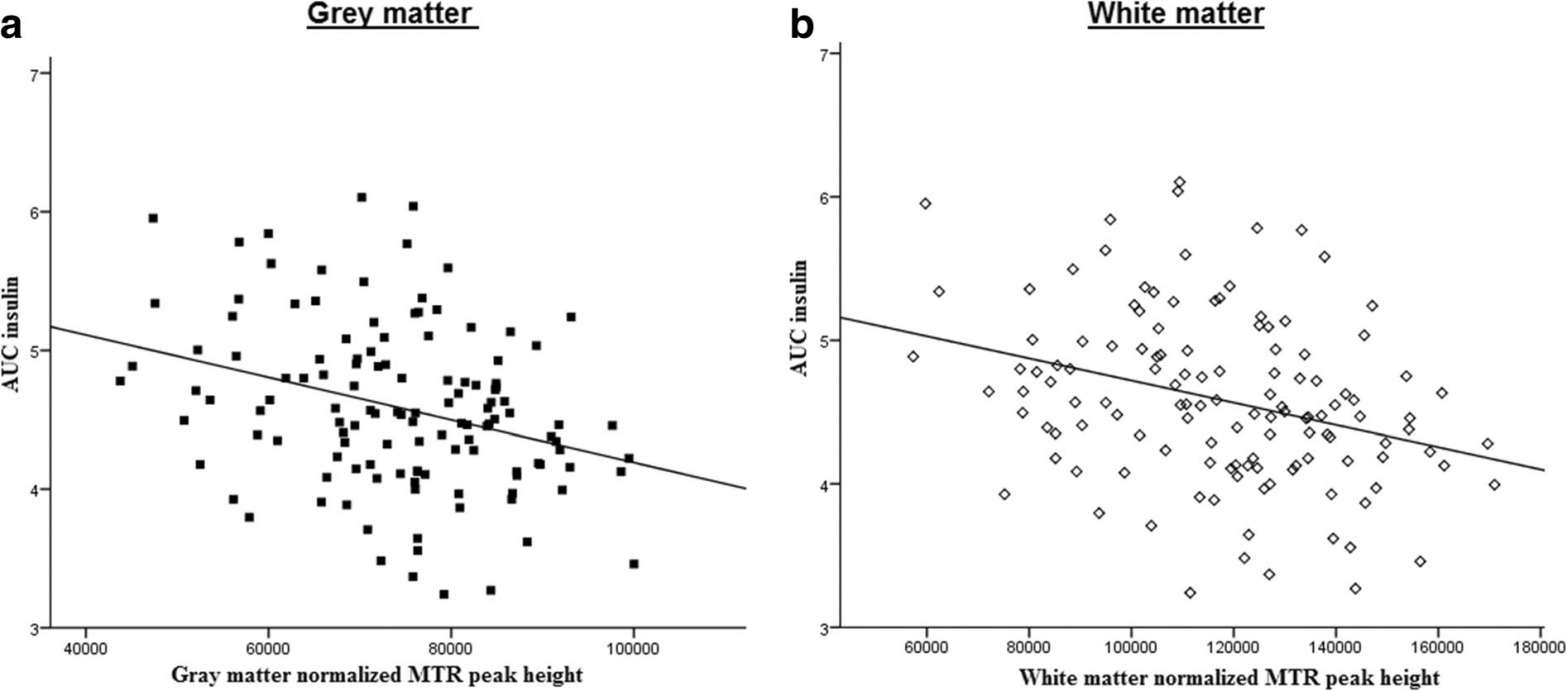
Relation between the area under the insulin curve and MTR peak height in gray and white matter. *Scatterplots* showing the inverse relation between area under the insulin curve (AUC_insulin_) and **a** gray matter and **b** white matter MTR peak height. *Lines of best fit* were derived from bivariate Pearson’s correlations

**Fig. 3:**
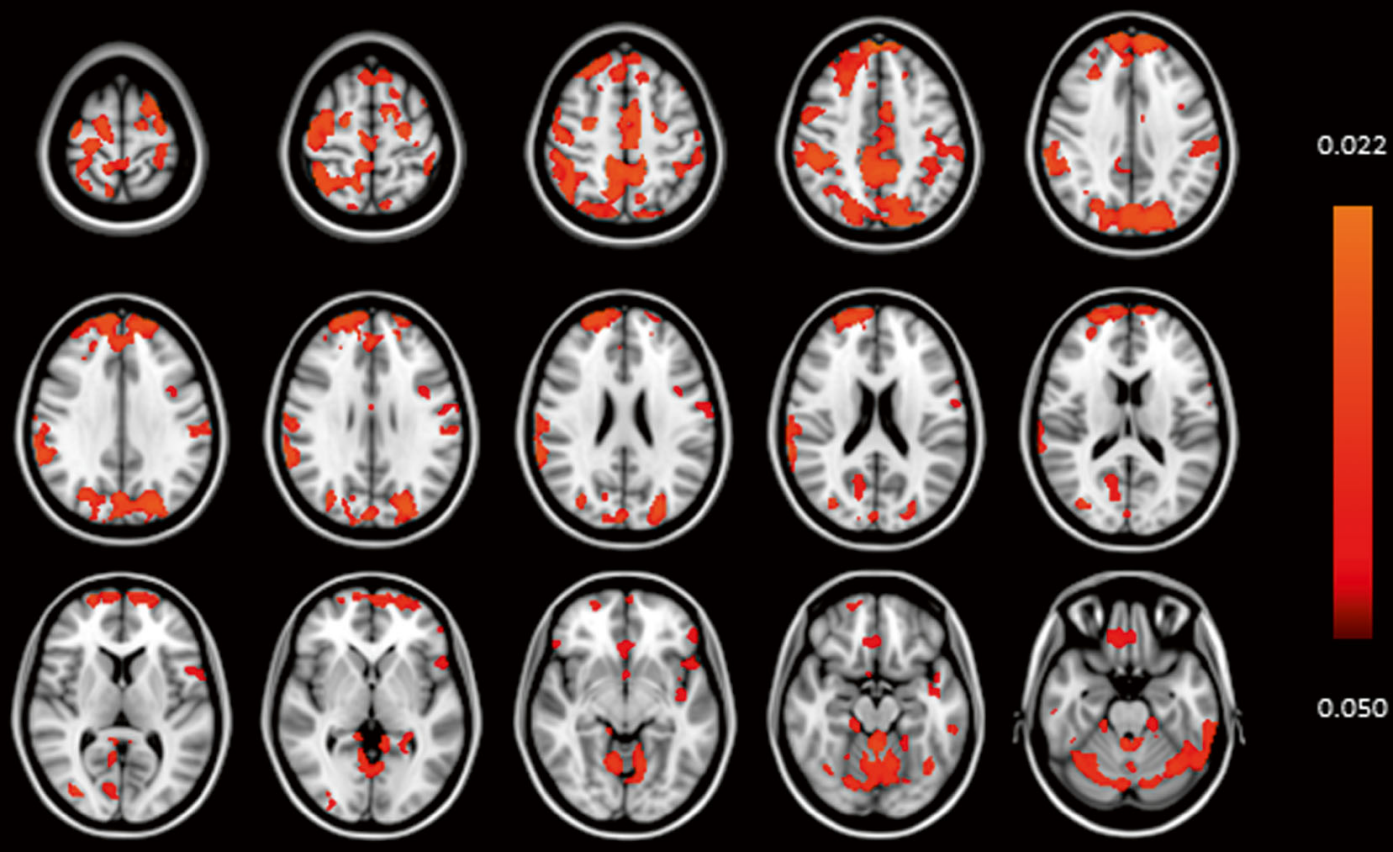
Voxel-based analysis of relation between cortical gray matter magnetization transfer ratio and insulin. Color scaling legend: color (*red-orange*) represents voxels that are statistically significant for lower gray matter MTR in association with higher AUC insulin (area under the insulin curve). Results are from voxel-based morphometric (VBM) analysis of cortical gray matter MTI magnetization transfer ratio (MTR). Results are projected on the MNI152 space T1-weighted image provided by FSL. Statistical analysis was adjusted for sex, age and offspring-partner status. Threshold-free cluster enhancement was applied with a significance level set at *P* < 0.05, corrected for family wise error rate

For visualization of the relations between reduced insulin action and brain micro-structure, scatterplots were made as well as voxel-based analysis, using area under the insulin curve (AUC_insulin_). Figure 2 shows the inverse relation of AUC_insulin_ with gray matter peak height (Fig. 2a) and white matter peak height (Fig. 2b). Furthermore, via voxel-based morphometric analysis, the associations between cortical gray matter MTR with AUC_insulin_ are projected on T1-weighted images, as shown in Fig. 3, where the corresponding decrease in cortical gray matter MTR with increasing AUC_insulin_ can be seen.

### Sensitivity analyses

To determine whether high ‘normal’ glucose levels, i. e. glucose levels in the upper normal range (5.7–6.8 mmol/l), modulated the observed effects of glucose, sub-analyses were also conducted with lower fasting glucose levels (<5.6 mmol/l). The findings of these were essentially similar to the results presented above.

## Conclusions and discussion

We report two main findings. The first is that subclinical variation in fasted glucose was associated with white matter atrophy. Secondly, in the absence of type 2 diabetes, higher insulin and reduced peripheral insulin action and sensitivity were associated with reduced micro-structural brain integrity in older adults without diabetes.

The role of metabolic derangement associated with (pre-) diabetic states in the decline in brain structure and function is an area of active investigation. Previous studies have shown relations between glucose levels (ranging from high normal to diabetic levels), with decreasing total brain volumes and cognition (Cui et al. 2014; Espeland et al. 2013; Mortby et al. 2013; Samaras et al. 2014). Associations between impairments in glucose regulation and smaller total brain volumes were found in a large cohort of middle-aged diabetic and non-diabetic subjects of the Framingham study, where inverse correlations were found between total cerebral brain volumes and HbA1c, HOMA-IR and fasted insulin levels (Tan et al. 2011). In harmony with these studies, we found a significant association between indices of impairments in glucose regulation (fasted glucose, fasted insulin, AUC_insulin_ and HOMA-IS) and gray and white matter micro-structure. Of note, there is an inverse relation between homeostatic model assessment of insulin sensitivity (HOMA-IS) and homeostatic model assessment of insulin resistance (HOMA-IR). The white matter atrophy observed in relation to fasted glucose in our study is consistent with previous research demonstrating high-‘normal’ blood glucose levels being associated with lower gray and white matter regional volumes (Mortby et al. 2013). Our findings thus suggest that the effect of plasma glucose on cerebral structural integrity in older people is not restricted to the upper normal range.

We describe here for the first time that increased fasted insulin and AUC_insulin_ and decreased HOMA-IS and insulinogenic index were significantly associated with decreased MTR histogram peak-height for both gray and white matter, indicating loss of homogeneity of brain tissue or tissue damage. Similar trends were found for mean MTR, but these were not statistically significant. Although mean MTR and histogram peak-height of MTR are both MTR measures, they reflect different aspects of MTR and may show considerable difference in sensitivity with respect to demonstrating variations in structural brain integrity (Benedetti et al. 2006; Ropele et al. 2010; van den Bogaard et al. 2012; Yamamoto et al. 2006). Peak heights of MTR histograms are a sensitive measure of micro-structural brain parenchymal abnormalities, loss of which has been associated with both aging and metabolic syndrome (Benedetti et al. 2006; Sala et al. 2014). Of note, previous population-based, longitudinal studies have suggested insulin and insulin resistance as being a link between diabetes and neurodegenerative diseases (Ronnemaa et al. 2008; Schrijvers et al. 2010). Interestingly, our findings showed lower MTR histogram peak heights in gray and white matter in relation with higher fasted insulin and decreasing sensitivity to insulin. We found an inverse association between fasted glucose and cerebral micro-bleeds, which was of borderline significance (*P* = 0.045). However, since the *R*^2^ was very low (*R*^2^ = 0.053), we could not exclude the possibility that this could have been a chance finding. No other significant association was found between indices of disturbances in glucose or insulin parameters and macro-structural brain pathology (white matter hyper-intensities, lacunar infarcts, cerebral micro-bleeds).

Taken together, these findings suggest that metabolically related brain structural abnormalities are observable at a microscopic level, even in the presence of glucose and insulin levels that are considered normal by present standards. An inverse association was found between OGTT-derived insulin parameters (fasted insulin, AUC_insulin_, HOMA-IS and insulinogenic index) and gray and white matter micro-structural integrity. This suggests a link between reduced insulin action (evidenced by higher insulin and reduced peripheral insulin sensitivity) and loss of homogeneity of brain tissue (reflecting parenchymal abnormalities), even in older adults without diabetes. One possible mechanism that may underpin these findings is that with aging, the ability to maintain the delicate balance between the various gluco-regulatory mechanisms declines, leading to deleterious micro-structural changes in neuronal and thus brain tissue integrity. Such micro-structural brain changes may exist even without the appearance of overt macro-structural changes that are associated with clinically significant metabolic disease.

It is a limitation of this study that we only examined cross-sectional associations and did not examine the association of changes of these measures over time. Thus, our findings are descriptive, and no causal inference can be made. A strength of this study is that, in addition to using conventional MRI, magnetization transfer imaging (MTI) was used, which is an advanced MRI technique that has the discriminatory power to detect in vivo micro-structural brain changes and quantitatively measure brain parenchyma abnormalities that are beyond the spatial resolution of conventional MRI.

In conclusion, using sensitive MRI techniques, we observed that subclinical differences in glucose and insulin metabolism were associated with macro- and micro-structural brain changes in older adults, and these were detectable even with glucose and insulin levels within population reference ranges. These findings possibly offer more insight into the association between different parameters of glucose metabolism and brain aging. Sufficiently powered follow-up studies are needed to evaluate cause or consequence in the relation between parameters of glucose metabolism and brain integrity.

## Abbreviations

AUC: Area under the curve
CMB: Cerebral micro-bleeds
FAST: FMRIB’s automated segmentation tool
FLAIR: Fluid attenuated inversion recovery
FMRIB: Functional MRI of the brain
FSL: FMRIB software library
GM: Gray matter
HOMA-IS: Homeostatic model assessment of insulin sensitivity
LLS: Leiden Longevity Study
MNI152: Montreal Neurological Institute 152
MRI: Magnetic resonance imaging
MTI: Magnetization transfer imaging
MTR: Magnetization transfer ratio
OGTT: Oral glucose tolerance test
SIENAX: Structural image evaluation, using normalization, of atrophy
TFCE: Threshold-free cluster enhancement
Type 2 DM: Type 2 diabetes mellitus
VOI: Voxels of interest
WM: White matter
WMH: White matter hyper-intensities

## References

[CR1] Altmann-Schneider I, de Craen AJ, Slagboom PE, Westendorp RG, van Buchem MA, Maier AB, van der Grond J (2012). Brain tissue volumes in familial longevity: the Leiden Longevity Study. Aging Cell.

[CR2] Andersson J, Jenkinson J, Smith S (2007) Non-linear registration aka spatial normalisation. FMRIB Technial Report TR07JA2 http://fmrib.medsci.ox.ac.uk/analysis/techrep/tr07ja2/tr07ja2.pdf

[CR3] Benedetti B, Charil A, Rovaris M, Judica E, Valsasina P, Sormani MP, Filippi M (2006). Influence of aging on brain gray and white matter changes assessed by conventional, MT, and DT MRI. Neurology.

[CR4] Biessels GJ, Staekenborg S, Brunner E, Brayne C, Scheltens P (2006). Risk of dementia in diabetes mellitus: a systematic review. Lancet Neurol.

[CR5] Bokura H, Kobayashi S, Yamaguchi S (1998). Distinguishing silent lacunar infarction from enlarged Virchow-Robin spaces: a magnetic resonance imaging and pathological study. J Neurol.

[CR6] Cosottini M (2011). Magnetization transfer imaging demonstrates a distributed pattern of microstructural changes of the cerebral cortex in amyotrophic lateral sclerosis. AJNR Am J Neuroradiol.

[CR7] Cowie CC (2006). Prevalence of diabetes and impaired fasting glucose in adults in the U.S. population: National Health and Nutrition Examination Survey 1999–2002. Diabetes Care.

[CR8] Crane PK (2013). Glucose levels and risk of dementia. N Engl J Med.

[CR9] Cui X, Abduljalil A, Manor BD, Peng CK, Novak V (2014). Multi-scale glycemic variability: a link to gray matter atrophy and cognitive decline in type 2 diabetes. PLoS One.

[CR10] de la Monte SM, Wands JR (2008). Alzheimer’s disease is type 3 diabetes-evidence reviewed. J Diabetes Sci Technol.

[CR11] Espeland MA (2013). Influence of type 2 diabetes on brain volumes and changes in brain volumes: results from the women’s health initiative magnetic resonance imaging studies. Diabetes Care.

[CR12] Greenberg SM (2009). Cerebral microbleeds: a guide to detection and interpretation. Lancet Neurol.

[CR13] Hanson RL (2000). Evaluation of simple indices of insulin sensitivity and insulin secretion for use in epidemiologic studies. Am J Epidemiol.

[CR14] Inglese M, Ge Y (2004). Quantitative MRI: hidden age-related changes in brain tissue. Top Magn Reson Imaging.

[CR15] Jenkinson M, Bannister P, Brady M, Smith S (2002). Improved optimization for the robust and accurate linear registration and motion correction of brain images. NeuroImage.

[CR16] Longstreth Jr WT, Bernick C, Manolio TA, Bryan N, Jungreis CA, Price TR (1998) Lacunar infarcts defined by magnetic resonance imaging of 3660 elderly people: the cardiovascular health study. Arch Neurol 55:1217–1225 10.1001/archneur.55.9.1217 9740116

[CR17] Matthews DR, Hosker JP, Rudenski AS, Naylor BA, Treacher DF, Turner RC (1985). Homeostasis model assessment: insulin resistance and beta-cell function from fasting plasma glucose and insulin concentrations in man. Diabetologia.

[CR18] Mortby ME, Janke AL, Anstey KJ, Sachdev PS, Cherbuin N (2013). High “normal” blood glucose is associated with decreased brain volume and cognitive performance in the 60s: the PATH through life study. PLoS One.

[CR19] Pruessner JC, Kirschbaum C, Meinlschmid G, Hellhammer DH (2003). Two formulas for computation of the area under the curve represent measures of total hormone concentration versus time-dependent change. Psychoneuroendocrinology.

[CR20] Rademacher J, Engelbrecht V, Burgel U, Freund H, Zilles K (1999). Measuring in vivo myelination of human white matter fiber tracts with magnetization transfer MR. NeuroImage.

[CR21] Ronnemaa E (2008). Impaired insulin secretion increases the risk of Alzheimer disease. Neurology.

[CR22] Ropele S, Enzinger C, Sollinger M, Langkammer C, Wallner-Blazek M, Schmidt R, Fazekas F (2010). The impact of sex and vascular risk factors on brain tissue changes with aging: magnetization transfer imaging results of the Austrian stroke prevention study. AJNR Am J Neuroradiol.

[CR23] Sala M (2014). Microstructural brain tissue damage in metabolic syndrome. Diabetes Care.

[CR24] Samaras K (2014). The impact of glucose disorders on cognition and brain volumes in the elderly: the Sydney memory and ageing study. Age (Dordr).

[CR25] Schoenmaker M, de Craen AJ, de Meijer PH, Beekman M, Blauw GJ, Slagboom PE, Westendorp RG (2006). Evidence of genetic enrichment for exceptional survival using a family approach: the Leiden Longevity Study. Eur J Hum Genet.

[CR26] Schrijvers EM, Witteman JC, Sijbrands EJ, Hofman A, Koudstaal PJ, Breteler MM (2010). Insulin metabolism and the risk of Alzheimer disease: the Rotterdam study. Neurology.

[CR27] Smith SM (2002). Fast robust automated brain extraction. Hum Brain Mapp.

[CR28] Smith SM, Nichols TE (2009). Threshold-free cluster enhancement: addressing problems of smoothing, threshold dependence and localisation in cluster inference. NeuroImage.

[CR29] Smith SM, Zhang Y, Jenkinson M, Chen J, Matthews PM, Federico A, De SN (2002). Accurate, robust, and automated longitudinal and cross-sectional brain change analysis. NeuroImage.

[CR30] Steen E (2005). Impaired insulin and insulin-like growth factor expression and signaling mechanisms in Alzheimer’s disease—is this type 3 diabetes?. J Alzheimers Dis.

[CR31] Stijntjes M (2013). Familial longevity is marked by better cognitive performance at middle age: the Leiden Longevity Study. PLoS One.

[CR32] Takeuchi M (2000). Evaluation of factors during OGTT to correlate insulin resistance in non-diabetic subjects. Endocr J.

[CR33] Tan ZS (2011). Association of metabolic dysregulation with volumetric brain magnetic resonance imaging and cognitive markers of subclinical brain aging in middle-aged adults: the Framingham Offspring Study. Diabetes Care.

[CR34] Vagelatos NT, Eslick GD (2013). Type 2 diabetes as a risk factor for Alzheimer’s disease: the confounders, interactions, and neuropathology associated with this relationship. Epidemiol Rev.

[CR35] van Buchem MA, Tofts PS (2000). Magnetization transfer imaging. Neuroimaging Clin N Am.

[CR36] van den Bogaard SJ (2012). Magnetization transfer imaging in premanifest and manifest Huntington disease. AJNR Am J Neuroradiol.

[CR37] Yamamoto A (2006). Whole brain magnetization transfer histogram analysis of pediatric acute lymphoblastic leukemia patients receiving intrathecal methotrexate therapy. Eur J Radiol.

[CR38] Zhang Y, Brady M, Smith S (2001). Segmentation of brain MR images through a hidden Markov random field model and the expectation-maximization algorithm. IEEE Trans Med Imaging.

